# Additional stimulation of sGC on top of standard treatment with ARB`s may offer a new therapeutic approach for the treatment of diabetic nephropathy resistant to ARB treatment alone

**DOI:** 10.1186/1471-2210-11-S1-P1

**Published:** 2011-08-01

**Authors:** Markus Alter, Ina Ott, Karoline von Websky, Oleg Tsuprykov, Yuliya Sharkovska, Katharina Krause-Relle, Jens Raila, Andrea Henze, Axel Kretschmer, Johannes-Peter Stasch, Berthold Hocher

**Affiliations:** 1 Department of Nephrology, Charité, Campus Benjamin Franklin, Berlin, Germany; 2 Center for Cardiovascular Research, Charité, Campus Mitte, Berlin, Germany; 3 Institute for Nutritional Science, University of Potsdam, Germany; 4 Bayer HealthCare AG, Cardiovascular Research, Wuppertal, Germany; 5 Institute of Pharmacy, Martin-Luther-University of Halle, Germany

## Background

Riociguat is the first of a new class of drugs, the soluble guanylate cyclase (sGC) stimulators. Riociguat has a dual mode of action: it sensitizes sGC to the body’s own NO and can also increase sGC activity in the absence of NO. The NO-sGC-pathway is impaired in many cardiovascular diseases such as heart failure, pulmonary hypertension and diabetic nephropathy (DN). DN leads to high cardiovascular morbidity and mortality. There is still a high unmet medical need. The urinary albumin excretion rate is a predictive biomarker for these clinical events. Therefore, we investigated the effect of riociguat, alone and in combination with the angiotensin II receptor antagonist (ARB) telmisartan on the progression of DN in diabetic eNOS knock out mice, a new model closely resembling human pathology.

## Methods

Seventy-six male eNOS knockout C57BL/6J mice were divided into 4 groups after receiving intraperitoneal high-dose streptozotocin: telmisartan (1 mg/kg), riociguat (3 mg/kg), riociguat+telmisartan (3 and 1 mg/kg), and vehicle. Fourteen mice were used as non-diabetic controls. After 12 weeks, urine and blood were obtained and blood pressure measured. Glucose concentrations were highly increased and similar in all diabetic groups.

## Results

Riociguat, alone (105.2 ± 2.5 mmHg; mean±SEM; n = 14) and in combination with telmisartan (105.0 ± 3.2 mmHg; n = 12), significantly reduced blood pressure versus diabetic controls (117.1 ± 2.2 mmHg; n = 14; p = 0.002 and p = 0.004, respectively), whereas telmisartan alone (111.2 ± 2.6 mmHg) showed a modest blood pressure lowering trend (p = 0.071; n = 14). The effects of single treatment with either riociguat (97.1 ± 15.7 µg/d; n = 13) or telmisartan (97.8 ± 26.4 µg/d; n = 14) did not significantly lower albumin excretion on its own (p = 0.067 and p = 0.101, respectively). However, the combined treatment led to significantly lower urinary albumin excretion (47.3 ± 9.6 µg/d; n = 12) compared to diabetic controls (170.8 ± 34.2 µg/d; n = 13; p = 0.004), and reached levels similar to non-diabetic controls (31.4 ± 10.1 µg/d, n = 12).

## Conclusion

Riociguat significantly reduced urinary albumin excretion in diabetic eNOS knock out mice that were refractory to treatment with ARB’s alone. Patients with diabetic nephropathy refractory to treatment with ARB’s have the worst prognosis among all patients with diabetic nephropathy. Our data indicate that additional stimulation of sGC on top of standard treatment with ARB`s may offer a new therapeutic approach for patients with diabetic nephropathy resistant to ARB treatment.

